# Supplementary material for: UBAP2L ensures homeostasis of nuclear pore complexes at the intact nuclear envelope
Source: J Cell Biol. 2024 Apr 23;223(7):e202310006. doi: 10.1083/jcb.202310006 (PMC11040503; doi:10.1083/jcb.202310006)
Supplement: Table S1 — describes the cloning primers used in the study. [file JCB_202310006_TableS1.docx]

**Table S1 Cloning primers**

| **Cloning of hUBAP2L in pEGFP-C1** | |
| --- | --- |
| pEGFP-C1-hUBAP2L-WT-Fwd | ttattaCTCGAGCCATGATGACATCGGTGGGCACTAACCG |
| pEGFP-C1-hUBAP2L-WT-Rvs | attattGAATTCTCAGTTGGCCCCCCAGCTGTAGC |
| pEGFP-C1-hUBAP2L-UBA (1-97 aa)-Fwd | ttattaCTCGAGCCATGATGACATCGGTGGGCACTAACCG |
| pEGFP-C1-hUBAP2L-UBA (1-97 aa)-Rvs | tatataGAATTCtcaCTCCCAGGAATGCGTGTCTGG |
| pEGFP-C1-hUBAP2L-98-430 aa-Fwd | ttattaCTCGAGCCatgGTCGGGAAGAAGAAGGGAGTC |
| pEGFP-C1-hUBAP2L-98-430 aa-Rvs | tatataGAATTCtcaGGTTGAAGATGGGGTAAAAGCC |
| pEGFP-C1-hUBAP2L-1-430 aa-Fwd | ttattaCTCGAGCCATGATGACATCGGTGGGCACTAACCG |
| pEGFP-C1-hUBAP2L-1-430 aa-Rvs | tatataGAATTCtcaGGTTGAAGATGGGGTAAAAGCC |
| pEGFP-C1-hUBAP2L-ΔUBA-Fwd | ttattaCTCGAGCCatgGTCGGGAAGAAGAAGGGAGTC |
| pEGFP-C1-hUBAP2L-ΔUBA-Rvs | attattGAATTCTCAGTTGGCCCCCCAGCTGTAGC |
| pEGFP-C1-hUBAP2L-Δ(UBA+RGG)-Fwd | ttattaCTCGAGCCatgGGAACCTTTAACCCAGCTG |
| pEGFP-C1-hUBAP2L-Δ(UBA+RGG)-Rvs | attattGAATTCTCAGTTGGCCCCCCAGCTGTAGC |
| pEGFP-C1-hUBAP2L-Δ1-429 aa-Fwd | tatattCTCGAGCCatgATGGAGGTGTTCCTTCAGGAG |
| pEGFP-C1-hUBAP2L-Δ1-429 aa-Rvs | attattGAATTCTCAGTTGGCCCCCCAGCTGTAGC |
| **Cloning of hFXR1 in pEGFP-C1** | |
| pEGFP-C1-hFXR1-WT-Fwd | ttattaCTCGAGCCATGGCGGAGCTGACGGTGGAGG |
| pEGFP-C1-hFXR1-WT-Rvs | tattatGAATTCTTATGAAACACCATTCAGGACTGC |
| **Cloning of Nup85 in pSNAPf-C1** | |
| pSNAPf-C1-hNup85-WT-Fwd (BamHI) | ataataGGATCCATGGAGGAGCTCGATGGCGAGCCAACAGTCACTTTGATTCCAGGCGTGAATTCCAAGAAGAACCAAATGTATTTTG |
| pSNAPf-C1-hNup85-WT-Rvs (XhoI) | taataaCTCGAGTCAGGAACCTTCCAGTGAGCCTTCTC |
| **Cloning of hUBAP2L in pcDNA3.1-Flag-N** | |
| pcDNA3.1-Flag-N-hUBAP2L-WT-Fwd | tttGAATTCTTATGACATCGGTGGGCACTAACC |
| pcDNA3.1-Flag-N-hUBAP2L-WT-Rvs | tttCTCGAGTCAGTTGGCCCCCCAGC |
| **Cloning of hUBAP2L KO sgRNAs in pX330-P2A-EGFP/RFP** | |
| hUBAP2L KO exon5 sgRNA-1-Fwd | caccGTGGCCAGACGGAATCCAATG |
| hUBAP2L KO exon5 sgRNA-1-Rvs | aaacCATTGGATTCCGTCTGGCCAC |
| hUBAP2L KO exon5 sgRNA-2-Fwd | caccGGTGGTGGGCCACCAAGACGG |
| hUBAP2L KO exon5 sgRNA-2-Rvs | aaacCCGTCTTGGTGGCCCACCACC |
| U6-CRISP/Cas9-promoter sequencing primer | gggcctatttcccatgattc |
| **Sequencing of UBAP2L KO clones - cloning of genomic DNA in pUC57** | |
| hUBAP2L KO exon5-DNA sequencing-Fwd | CGAATGCATCTAGATATCGGATCCCTGCTGAGTGGAGAATGGTTA |
| hUBAP2L KO exon5-DNA sequencing-Rvs | GCCTCTGCAGTCGACGGGCCCGGGAGACTGGTGGCAGTTGGTAG |
