# Supplementary material for: UBAP2L ensures homeostasis of nuclear pore complexes at the intact nuclear envelope
Source: J Cell Biol. 2024 Apr 23;223(7):e202310006. doi: 10.1083/jcb.202310006 (PMC11040503; doi:10.1083/jcb.202310006)
Supplement: Table S2 — describes other reagents and resources including bacterial stains, cell lines, chemicals, cDNAs, and software used in the study. [file JCB_202310006_TableS2.docx]

**Table S2 Reagents and resources**

| REAGENT or RESOURCE | SOURCE | IDENTIFIER |
| --- | --- | --- |
| **Bacterial strains** | | |
| DH5alpha Competent *E. coli* | NEW ENGLAND BioLabs | Cat# C2987I |
| **Chemicals and Peptides** | | |
| Thymidine | Sigma-Aldrich | Cat# T1895-5G |
| Nocodazole | Sigma-Aldrich | Cat# M-1404 |
| Monastrol | Sigma-Aldrich | Cat# M8515 |
| 4′,6-Diamidino-2-phenylindole dihydrochloride (DAPI) | Sigma-Aldrich | Cat# D8417 |
| MG132 | Tocris Bioscience | Cat# 1748 |
| STLC (S-Trityl-L-cysteine) | Enzo Life Sciences | Cat# ALX-105-011-M500 |
| MOWIOL 4-88 Reagent | Millipore | Cat# **475904-M** |
| jetPEI^®^-DNA transfection reagent | Polyplus transfection | Cat# 101-01N |
| SiR-DNA | Sprirochrom | Cat# SC007 |
| Lipofectamine™ 2000 Transfection Reagent | Invitrogen | Cat# 11668019 |
| Lipofectamine™ RNAiMAX Transfection Reagent | Invitrogen | Cat# 13778150 |
| X-tremeGENE™ 9 DNA Transfection Reagent | Roche | Cat# 6365787001 |
| Dexamethasone | Sigma-Aldrich | Cat# D8833 |
| T4 DNA Ligase | New England Biolabs | Cat# M0202T |
| SNAP-Cell TMR-Star | New England Biolabs | Cat# S9105S |
| Exonuclease III | Takara | Cat# 2170B |
| Cycloheximide | Sigma-Aldrich | Cat# C4859 |
| Glucose oxidase | Sigma-Aldrich | Cat# G2133 |
| Cyclooctatetraene | Sigma-Aldrich | Cat# 138924 |
| Catalase | Sigma-Aldrich | Cat# C1345 |
| Leptomycin B | Abcam | Cat# ab120501 |
| Lovastatin | Sigma-Aldrich | Cat# 75330-75-5 |
| Psoralidin | Sigma-Aldrich | Cat# 18642-23-4 |
| Benzonase® Nuclease | Millipore | Cat# 70746 |
| RNAse A | Thermo Fisher Scientific | Cat# EN0531 |
| Propidium iodide (PI) | Sigma-Aldrich | Cat# P4170 |
| Phalloidin 488 | Thermo Fisher Scientific | Cat# A12379 |
| **Cell Lines** | | |
| Human: HeLa (Kyoto) | ATCC | Cat# CCL-2 |
| Human: HeLa UBAP2L KO | (Guerber et al, 2023) | N/A |
| Human: HeLa SNAP-Nup85 | This study | N/A |
| Human: U2OS bone osteosarcoma | ATCC | Cat# HTB-96 |
| Human: Nup96-GFP KI U2OS | (Thevathasan et al., 2019) | N/A |
| Human: Nup96-GFP KI U2OS UBAP2L KO | This study | N/A |
| **Oligonucleotides** | | |
| siRNA: Non-targeting siGENOME | Dharmacon | Cat# D-001210-02-05 |
| siRNA: FXR1 individual | Dharmacon | Cat# J-012011-06-0005 |
| siRNA: UBAP2L individual | Dharmacon | Cat# J-021220-09-0002 |
| Primers used for Cloning and Sequencing are described in Table S1 | This study | N/A |
| **Recombinant DNA** | | |
| pcDNA3.1-Flag-N | This study | N/A |
| pcDNA3.1-Flag-N-UBAP2L WT | This study | N/A |
| pcDNA3.1-Flag-UBAP2L R131-190A | (Huang et al., 2020) | N/A |
| pEGFP-C1 | Clontech | Cat# 6084-1 |
| pEGFP-C1-UBAP2L WT | This study | N/A |
| pEGFP-C1-UBAP2L UBA | This study | N/A |
| pEGFP-C1-UBAP2L ΔUBA | This study | N/A |
| pEGFP-C1-UBAP2L 98-430aa | This study | N/A |
| pEGFP-C1-UBAP2L 1-430aa | This study | N/A |
| pEGFP-C1-UBAP2L Δ1-429aa | This study | N/A |
| pEGFP-C1-UBAP2L Δ(ΔUBA+RGG) | This study | N/A |
| pEGFP-C1-FXR1 WT | This study | N/A |
| pSNAPf-C1 | Addgene | Cat# 58186 |
| pSNAPf-C1-hNup85 | This study | N/A |
| pEGFP-C1-Nup85 WT | (Loïodice et al., 2004) | N/A |
| pXRGG-GFP | (Hamada et al., 2011; Love et al., 1998) | N/A |
| pUC57 | Thermo | Cat# SD0171 |
| pX330-P2A-EGFP | (Zhang et al., 2017) | N/A |
| pX330-P2A-RFP | (Zhang et al., 2017) | N/A |
| **Software and Algorithms** | | |
| CRISPR/Cas9 Guide RNA Design | Benchling | https://www.benchling.com/ |
| Fiji Image Analysis | ImageJ | https://imagej.net/Fiji |
| Colocalization Analysis | CellProfiler | https://cellprofiler.org/ |
| Prism | GraphPad | N/A |
| Illustrator | Adobe | N/A |
| MATLAB | Mathworks | N/A |
