# Supplementary figures and images for: UBAP2L ensures homeostasis of nuclear pore complexes at the intact nuclear envelope
Source: J Cell Biol. 2024 Apr 23;223(7):e202310006. doi: 10.1083/jcb.202310006 (PMC11040503; doi:10.1083/jcb.202310006)

Corresponding to Fig. 2A

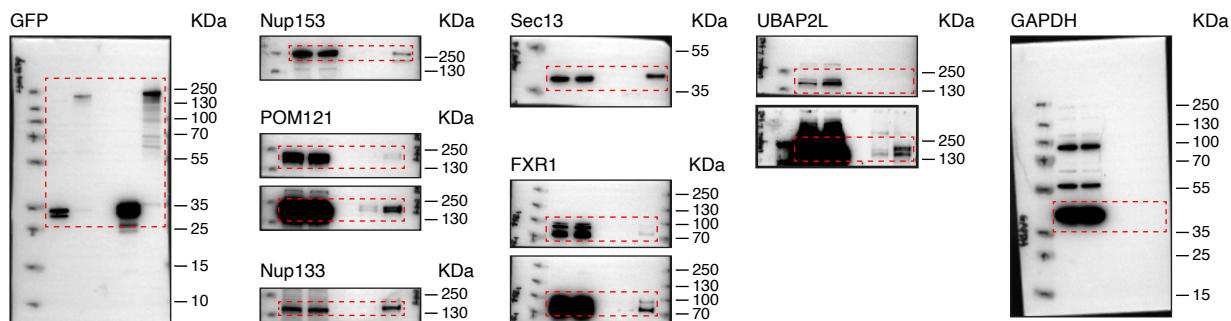

Corresponding to Fig. 2B

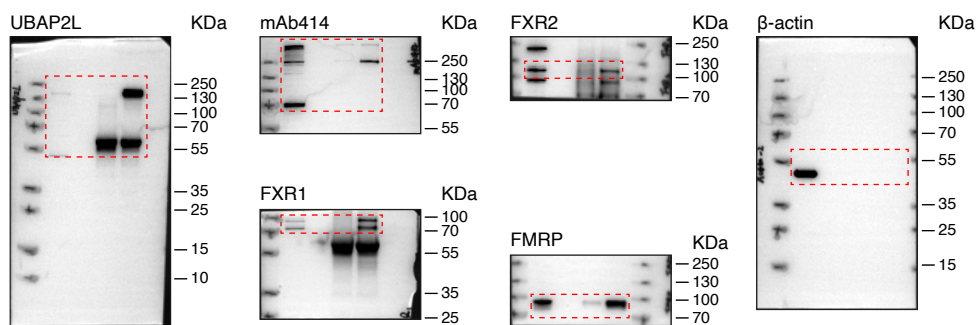

Corresponding to Fig. 2C

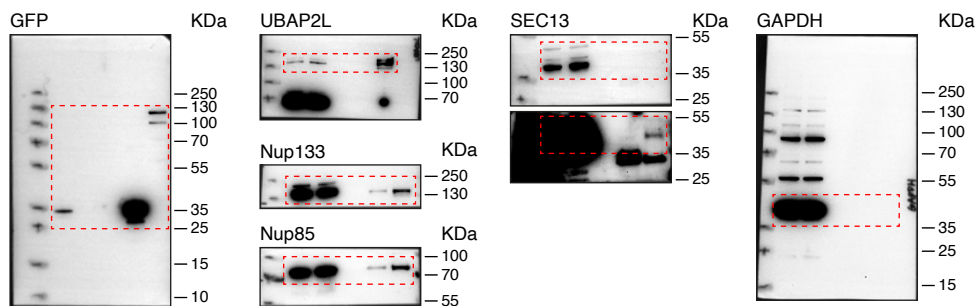

Supplement: SourceData F2 — is the source file for Fig. 2. [file JCB_202310006_SourceDataF2.pdf]

Corresponding to Fig. 3G

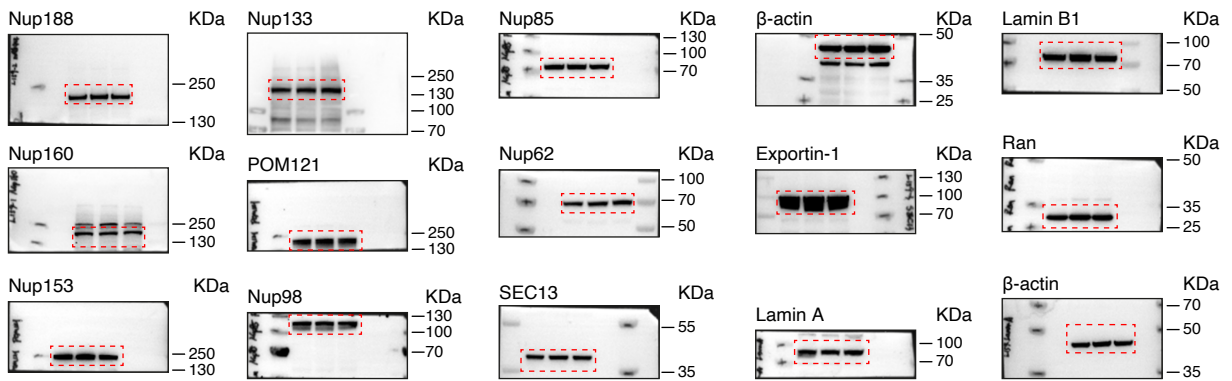

Corresponding to Fig. 3J

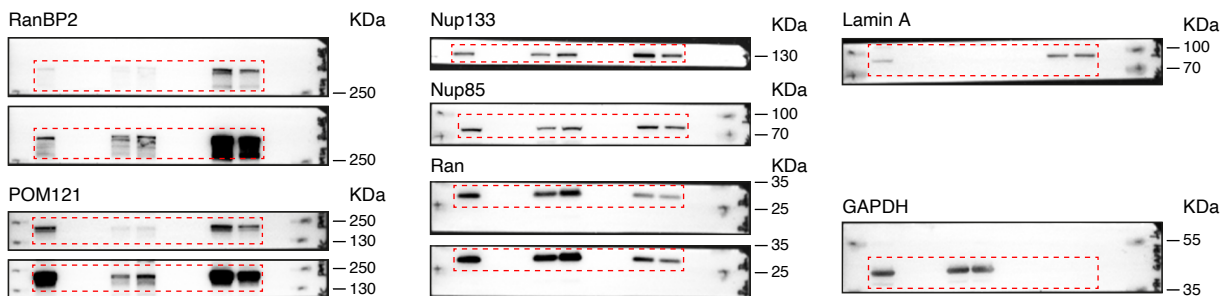

Supplement: SourceData F3 — is the source file for Fig. 3. [file JCB_202310006_SourceDataF3.pdf]

Corresponding to Fig. 5G

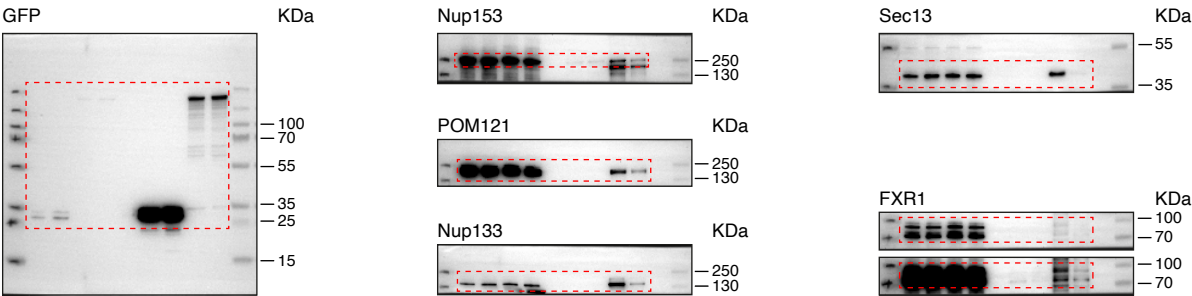

Corresponding to Fig. 5H

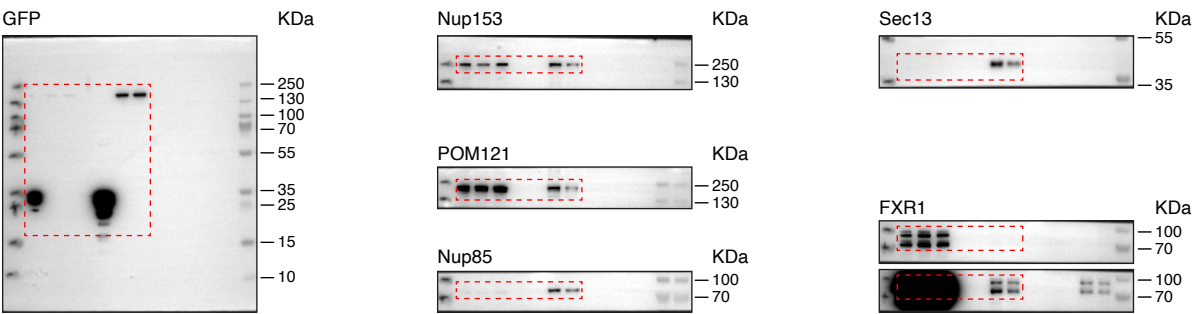

Supplement: SourceData F5 — is the source file for Fig. 5. [file JCB_202310006_SourceDataF5.pdf]

Corresponding to Fig. 6A

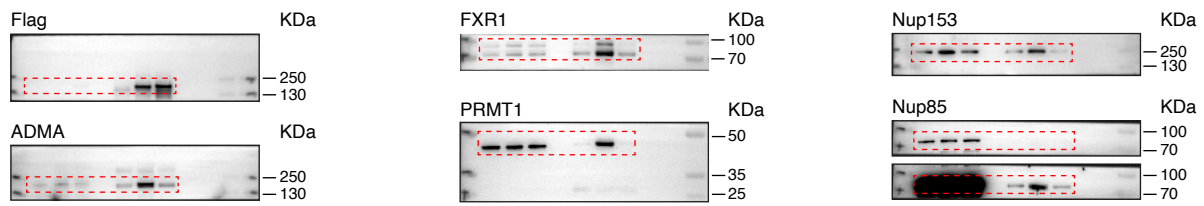

Supplement: SourceData F6 — is the source file for Fig. 6. [file JCB_202310006_SourceDataF6.pdf]

Corresponding to Fig. 9D

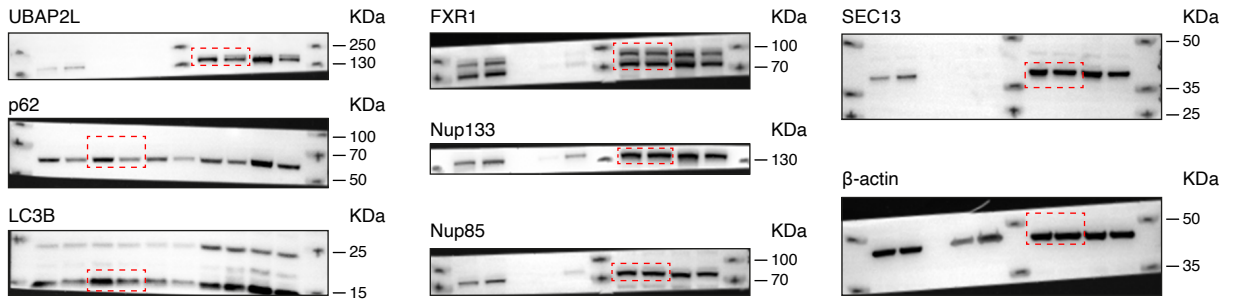

Supplement: SourceData F9 — is the source file for Fig. 9. [file JCB_202310006_SourceDataF9.pdf]

Corresponding to Fig. 10B

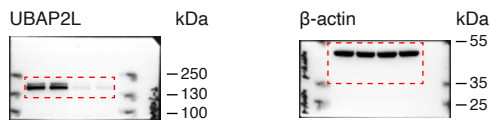

Corresponding to Fig. 10E

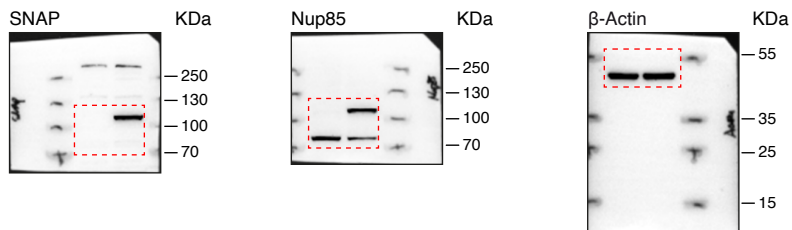

Corresponding to Fig. 10I

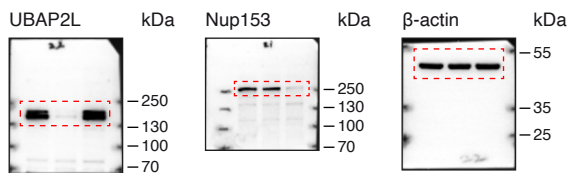

Supplement: SourceData F10 — is the source file for Fig. 10. [file JCB_202310006_SourceDataF10.pdf]

Corresponding to Fig. S1F

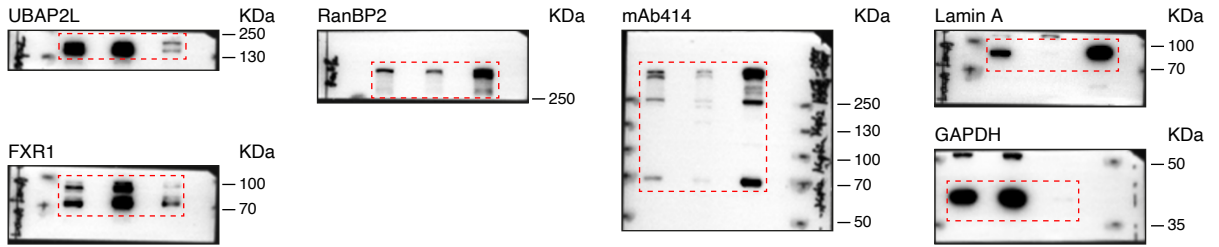

Supplement: SourceData FS1 — is the source file for Fig. S1. [file JCB_202310006_SourceDataFS1.pdf]
