# Supplementary material for: UBAP2L ensures homeostasis of nuclear pore complexes at the intact nuclear envelope
Source: J Cell Biol. 2024 Apr 23;223(7):e202310006. doi: 10.1083/jcb.202310006 (PMC11040503; doi:10.1083/jcb.202310006)

SourceDataFS2

Corresponding to Fig. S2D

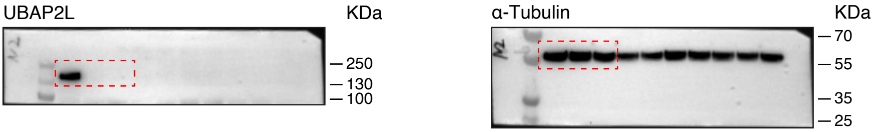

Corresponding to Fig. S2I

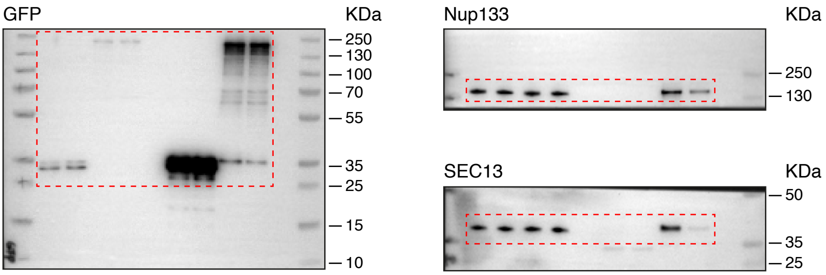

Corresponding to Fig. S2J

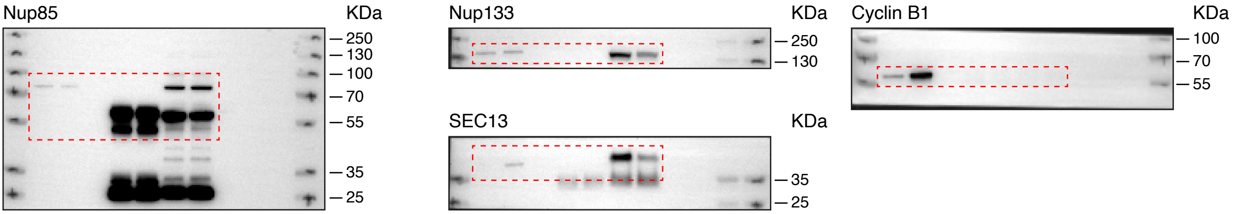

Corresponding to Fig. S2K

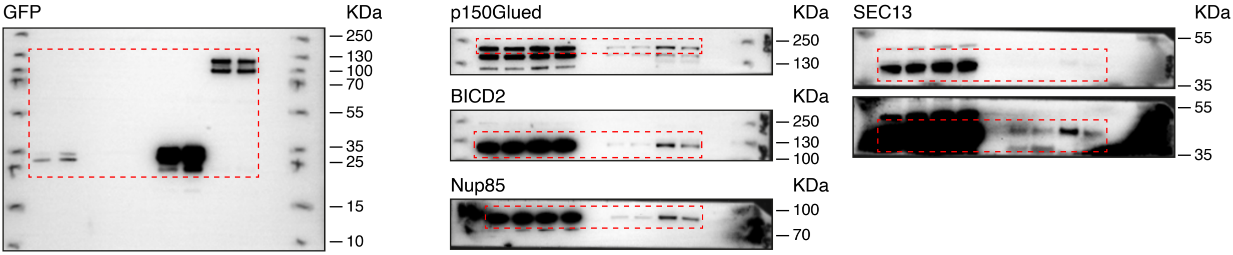

Supplement: SourceData FS2 — is the source file for Fig. S2. [file JCB_202310006_SourceDataFS2.pdf]
