# Supplementary material for: UBAP2L ensures homeostasis of nuclear pore complexes at the intact nuclear envelope
Source: J Cell Biol. 2024 Apr 23;223(7):e202310006. doi: 10.1083/jcb.202310006 (PMC11040503; doi:10.1083/jcb.202310006)

SourceDataFS3

Corresponding to Fig. S3F

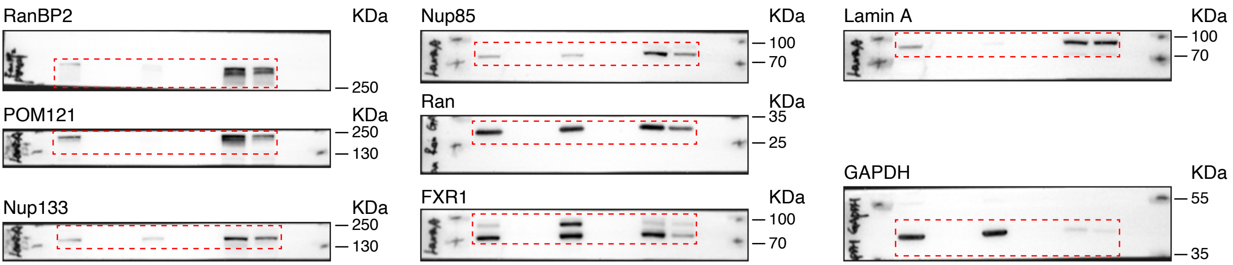

Corresponding to Fig. S3G

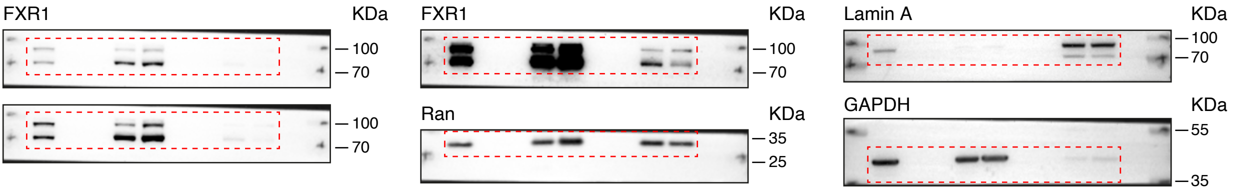

Corresponding to Fig. S3J

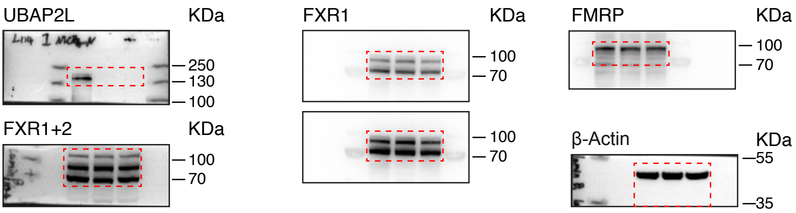

Corresponding to Fig. S3L

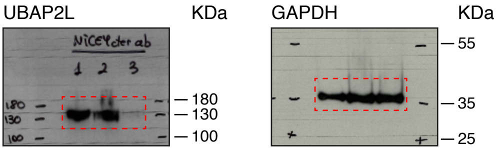

Supplement: SourceData FS3 — is the source file for Fig. S3. [file JCB_202310006_SourceDataFS3.pdf]
